# Development of Conformation Independent Computational Models for the Early Recognition of Breast Cancer Resistance Protein Substrates

**DOI:** 10.1155/2013/863592

**Published:** 2013-08-01

**Authors:** Melisa Edith Gantner, Mauricio Emiliano Di Ianni, María Esperanza Ruiz, Alan Talevi, Luis E. Bruno-Blanch

**Affiliations:** ^1^Medicinal Chemistry, Department of Biological Sciences, Faculty of Exact Sciences, National University of La Plata (UNLP), Argentinean National Council for Scientific and Technical Research (CONICET), CCT La Plata, Buenos Aires, B1900AJI La Plata, Argentina; ^2^Quality Control of Medications, Department of Biological Sciences, Faculty of Exact Sciences, National University of La Plata (UNLP), Buenos Aires, B1900AJI La Plata, Argentina; ^3^Biopharmacy, Department of Biological Sciences, Faculty of Exact Sciences, National University of La Plata (UNLP), 47 and 115, Buenos Aires, B1900AJI La Plata, Argentina

## Abstract

ABC efflux transporters are polyspecific members of the ABC superfamily that, acting as drug and metabolite carriers, provide a biochemical barrier against drug penetration and contribute to detoxification. Their overexpression is linked to multidrug resistance issues in a diversity of diseases. Breast cancer resistance protein (BCRP) is the most expressed ABC efflux transporter throughout the intestine and the blood-brain barrier, limiting oral absorption and brain bioavailability of its substrates. Early recognition of BCRP substrates is thus essential to optimize oral drug absorption, design of novel therapeutics for central nervous system conditions, and overcome BCRP-mediated cross-resistance issues. We present the development of an ensemble of ligand-based machine learning algorithms for the early recognition of BCRP substrates, from a database of 262 substrates and nonsubstrates compiled from the literature. Such dataset was rationally partitioned into training and test sets by application of a 2-step clustering procedure. The models were developed through application of linear discriminant analysis to random subsamples of Dragon molecular descriptors. Simple data fusion and statistical comparison of partial areas under the curve of ROC curves were applied to obtain the best 2-model combination, which presented 82% and 74.5% of overall accuracy in the training and test set, respectively.

## 1. Introduction 

ATP-binding cassette (ABC) efflux transporters comprise a diversity of active carriers which provide an efficient mechanism of defense against foreign chemicals (i.e., xenobiotics), among them drugs. To elicit the therapeutic response, drugs must often cross a number of cellular barriers, such as the gut wall and the capillaries endothelial cells. ABC efflux transporters limit drug absorption and distribution by translocating drugs from the cytoplasm to the cell exterior. These transporters are preferentially expressed at tissues that present barrier and/or excretory functions, for example, the intestinal wall, the canalicular membrane of hepatocytes in the liver, or the luminal membrane of the tubular cells in the kidney [1, 2], reducing the bioavailability of their substrates. Moreover, due to their wide substrate specificity, overexpression of such transporters is associated with cross-resistance phenomena to structurally unrelated drugs (multidrug resistance) in a wide range of diseases, from cancer to epilepsy [3–5]. Efflux transporters and metabolic enzymes seem to act in a coordinated or synergic manner, with the biotransformation products being often substrates for these drug carriers [1]. Furthermore, metabolizing enzymes and efflux transporters are also upregulated in a coordinated manner by common nuclear receptors that, upon environmental chemical triggering agents, induce the expression of host defense systems towards potentially toxic chemical agents [1, 6, 7]. 

Even though P-glycoprotein (also known as ABCB1 or MDR1) was the first identified and is the most extensively studied member of the ABC superfamily, recent studies suggest that the effect of another member, breast cancer resistance protein (BCRP, or ABCG2) might have been underestimated in the past. A number of reports indicate that BCRP is the most abundantly expressed ABC efflux transporter in different segments of human intestine, both at mRNA [8, 9] and protein levels [10]. Similar observations have been found at the blood-brain barrier, where BCRP mRNA levels are around 8 times above those of P-glycoprotein and represent 85% of the total ABC transporters mRNA [11], while at the protein level, BCRP levels are about 1.6 times higher [12, 13]. Therefore, regulation of BCRP and/or early recognition of BCRP substrates are critical aspects to optimize oral drug absorption, increase drug bioavailability, and design novel therapeutics aimed at brain conditions and diseases linked to BCRP-mediated multidrug resistance issues (e.g., cancer). 

The most advanced research regarding ABC transporters modulation relates to add-on therapies of specific inhibitors of ABC transporters, a strategy that was originally conceived for cancer treatment. Although preclinical and initial clinical results with first- and second-generation inhibitors have been encouraging, some trials stopped at phase III due to serious adverse effects [3, 5, 14–16]; such outcome has put in doubt the strategy of overcoming cellular drug resistance by the use of transporters inhibitors, even though trials continue in order to find more effective and safe inhibitors for P-glycoprotein and other drug carriers [16]. It is worth highlighting that ABC transporters comprise a concerted, complex transport system whose substrates are not only drugs, but also endogenous compounds (e.g., waste products, bile salts) and toxins. Thus, their permanent impairment or disruption is likely to result in severe side effects (especially in those therapeutic backgrounds that demand long-term treatment). Recent research has then focused on elucidating intracellular signaling pathways that control ABC transporters (their expression, intracellular trafficking, activation, and inactivation). It is proposed that finding the molecular switches of these transporters will allow selective modulation of transporters function and/or expression for therapeutic purposes in different clinical scenarios [17], which includes turning off the efflux mechanisms for short, controlled periods of time. Other alternatives, probably safer approaches propose avoidance of substrate-transporter interaction by the encapsulation of therapeutic agents within nanosystems (a “Trojan horse” approach) [18], or designing drugs or prodrugs which are not recognized by drug carriers [16, 19, 20]. 

At present, limited studies have been done to develop high throughput *in silico *models for the early identification of BCRP substrates, in order to assist virtual screening and computer-aided design of novel BCRP nonsubstrates therapeutics. Recently, Hazai et al. obtained a support vector machine model based on 5 Dragon descriptors [21]. To that purpose, they compiled a 263-compound wild-type BCRP substrates and nonsubstrates dataset which was randomly partitioned into a 167-compound unbalanced training set (it contained far more substrates than nonsubstrates), a 56-compound test set, and a 40-compound independent external set. The model showed an overall accuracy of 76% on the training set, 75% on the test set, and 72.5% on the external set; moreover, it presented much more accuracy in the identification of substrates than nonsubstrates (a possible consequence of the unbalanced training set). Some of the descriptors incorporated into this model were 3D (conformation dependent) structural features, which implies that considerable preprocessing (conformational analysis) of the predicted structures is needed before proceeding to prediction itself, which may hamper the screening efficiency of the algorithm, especially if we take into account that available public databases for virtual screening purposes (e.g., Drugbank, ZINC database) compile thousands to millions of chemical compounds. This same issue can be envisaged for the 17-descriptor model reported by Zhong et al. [22], who combined genetic algorithms and support vector machines to obtain, from a more limited unbalanced 177-compound dataset of BCRP substrates and nonsubstrates (again, randomly partitioned into training and test sets), a model with 85% overall accuracy. Once more, this model is majorly composed of 3D descriptors. 

It has been pointed out that the polyspecificity/broad substrate specificity of ABC transporters due to multiple separate binding sites or “binding zones,” binding sites accommodating more than one ligand and high protein flexibility determine a complex phenomenon which can only be partially addressed by current methods in the computational drug design field [23, 24]. This explains why many modeling efforts to identify ABC transporters substrates have resorted to ensemble learning or locally weighted methods [25–28]. In fact, BCRP presents at least two binding sites [29–31]. Here, we present the development of an ensemble of linear classificatory models capable of differentiating BCRP substrates and nonsubstrates. 

Contrasting the previously discussed models, our ensemble is entirely based on conformation independent descriptors, which makes it an adequate *in silico *filter to assist virtual screening campaigns in a highly efficient manner. The models have been derived from a relatively large 262-compound dataset which was rationally partitioned—through combined hierarchical and *k*-means clustering—into a representative and balanced 164-compound training set (85 substrates and 79 nonsubstrates) and a 98-compound test set (71 substrates and 27 nonsubstrates). Furthermore, on the basis of receiving operating characteristic (ROC) curves analysis, the score threshold can be optimized to prioritize the accuracy in the prediction of either substrates or nonsubstrates, depending on background-dependent criteria. In order to minimize the early selection of BCRP substrates as drug candidates, we have prioritized substrate accuracy. Such decision was supported by statistical comparison of the partial area under the curve (AUC) of ROC curves.

## 2. Materials and Methods

### 2.1. Dataset

**Figure 1:**
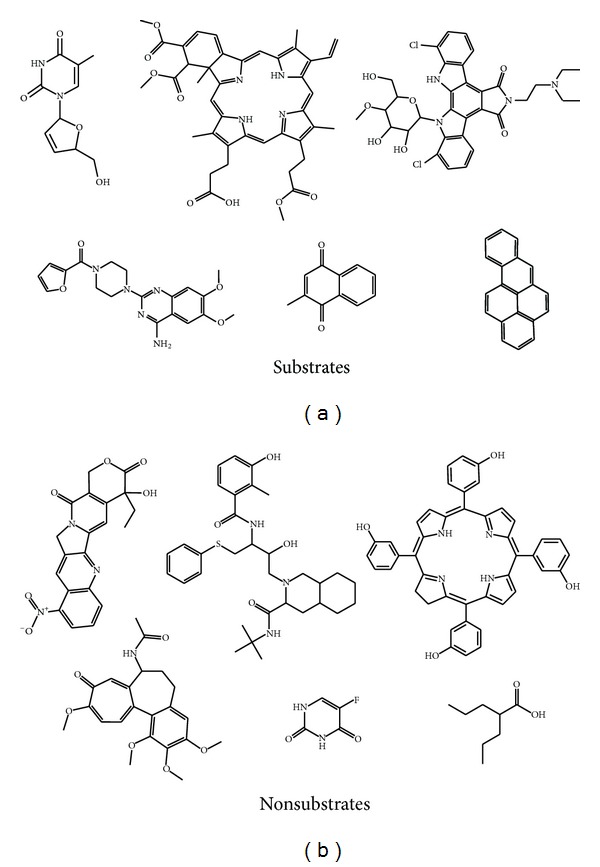
Representative BCRP substrates (left) and nonsubstrates (right) from the six most populated clusters in the dataset.

A 305-compound diverse dataset containing BCRP substrates and nonsubstrates was compiled from the literature. It is known that a single-nucleotide substitution at R482 can modify the affinity of BCRP for substrates [32–39]; however, the clinical consequences of such variant are not clear to the moment [40]. Therefore, from the original 305-compound dataset, we selected 262 compounds which are substrates and nonsubstrates of human wild-type BCRP, the subject of this modeling effort. BCRP substrates or nonsubstrates of BCRP homologs from other species with no evidence of human BCRP-mediated transport were not included to avoid noise due to inter-species variability in substrate specificity. The dataset was split into a 164-compound balanced training set (85 substrates, 79 nonsubstrates) and a 98-compound independent test set reserved for external validation. In order to obtain representative partitions of the dataset compounds, a combined hierarchical and *k*-means clustering approach was applied. The LibraryMCS v0.7 (ChemAxon) hierarchical clustering approach was applied in combination with the *k*-means clustering as implemented in Statistica 10 cluster analysis module (Statsoft Inc., 2011). LibraryMCS relies on the maximum common substructure (MCS, i.e., the largest subgraph shared by two chemical graphs) to cluster a set of chemical structures. The algorithm applies similarity search to the pool of molecules, and the two structures with the highest similarity coefficient are considered more likely to share a large MCS. Once this probable MCS has been established, substructure search is carried out in order to find the MCS of multiple structures efficiently, without exhaustive pairwise comparison. Certainly, it is possible that the two structures with highest similarity coefficient are not the ones that share the largest MCS; thus, library MCS leads to reproducible but approximate solutions [41]. As suggested by Everitt et al. [42], hierarchical clustering has been applied here to define an initial partition of *n* objects into *g* groups, selecting the smallest common substructure of 9 atoms. The groups of compounds were later optimized by *k*-means algorithm, minimizing the Euclidean distance to the group centers. A series of descriptors computed with Dragon 4.0 (Milano Chemometrics, 2003) representing different aspects of molecular structure (namely, molecular weight, log *P*, polar surface area, number of H bonds acceptors and donors, total information index of atomic composition, sum of atomic van der Waals volumes, sum of atomic Sanderson electronegativities, and 2D Petitjean shape index) were normalized and applied to calculate such distance. Once the clusters were separately identified in the substrates and nonsubstrates classes, around 50% of each cluster from the substrates category and 25% of each cluster in the nonsubstrates category were assigned to an independent test set for validation purposes, while the remaining percentage of the clusters was retained as training set for modeling purposes. This scheme allowed obtaining a balanced training set where, unlike in previous modeling efforts, neither the substrates nor the nonsubstrates were markedly overrepresented. The structures of both training and test set compounds are provided as pdf files in Supplementary information available online at http://dx.doi.org/10.1155/2013/863592 so that the reader can appreciate the structural diversity of the dataset. Representative members of each cluster in the substrate and non-substrate categories are shown in Figure 1.

### 2.2. Molecular Descriptor Calculation and Modeling Method

Dragon software for molecular descriptors calculation, version 4.0 (Milano Chemometrics, 2003) was used for the calculation of 867 low-dimensional (0D–2D) descriptors, distributed along 12 blocks of descriptors, for example, constitutional descriptors, topological descriptors, connectivity indices, Galvez topological charge indices, functional groups count, and others. Since such a high number of descriptors may result in chance correlations between the modeled property and a subset of descriptors, 102 subsets of descriptors obtained from random combinations of the blocks of Dragon low-dimensional descriptors were considered as independent pools of descriptors, each combination containing around 200 molecular descriptors. For example, the first pool of descriptors (180 descriptors) emerged from combination of the following Dragon blocks of descriptors: constitutional descriptors, eigenvalue-based indices, 2D autocorrelations and molecular properties; the second pool of descriptors (195 descriptors) combined walk and path counts, connectivity indices, functional group counts and BCUT descriptors, and so on. The use of this strategy, called *random subspace*, has proved to be effective for ensemble learning to combine weak learners in order to obtain strong learners [43, 44]. Descriptors with constant or near-constant values for the training set associated to low information content were removed from descriptors pools.

A binary, dummy variable codifying the category of each compound was used as dependent variable (class = 1 for substrates and class = −1 for nonsubstrates). Stepwise forward multiple linear regression was used to select the descriptors from each random pool that best discriminated the category of the compounds. Obtaining all possible descriptors subsets would demand *D*!/[*d*!(*D* − *d*)!], where *D* is the number of descriptors in a given descriptor pool and *d* is the number of descriptors included in a given model. This is very computationally demanding or even unfeasible when *D* is large, as in the present work. Therefore, we resort to a stepwise approach which, although faster, leads to suboptimal solutions. 

Linear discriminant analysis (LDA) was used to characterize the correspondent linear discriminant functions (dfs). Dfs assume the following general form:

(1) $$\begin{array}{c} \text{df value}=a_{0}+\sum_{i}a_{i}-d_{i}, \end{array}$$

where *a*_0_ is constant and *a*_*i*_ is the coefficient associated with molecular descriptor *d*_*i*_. Due to the values arbitrarily assigned to substrates and nonsubstrates, substrates will tend to have positive df values, and nonsubstrates will tend to assume negative values.

The binary classification scheme reduces the error associated with combining data obtained in different labs and conditions [45]. Multiple regression and discriminant analysis modules from Statistica 10 were used for modeling purposes. Tolerance values no lower than 0.1 were used in order to avoid inclusion of highly correlated pairs of descriptors. The minimum cases to predictors ratio allowed was 11 (11 or more cases in the training set for each descriptor included in the model) in order to reduce chances of overfitting; thus, models including at most between 10 and 15 descriptors were obtained through a stepwise forwards procedure. Only descriptors with significant coefficients at an alpha level of 0.05 are allowed into the model. Randomization, stratified leave-group-out (LGO) cross-validation and external validation (predicting the class for the independent 98-compound test set) were used to assess all models robustness and predictive ability. 50 randomized models were built in the randomization test. In each LGO row, 10 compounds were randomly removed from the training set, and the resulting LGO models were used to assess the category of the removed compounds; this process was repeated 50 times, checking that all the compounds in the training set had been removed in at least one LGO round. 

### 2.3. Combining Models

Two important indicators of the performance of a given QSAR model are sensitivity (Se) and specificity (Sp). They are defined by the following expressions:

(2) $$\begin{array}{c} \text{Se}=\frac{\text{TP}}{\text{TP}+\text{FN}},\\ \text{Sp}=\frac{\text{TN}}{\text{TN}+\text{FP}}, \end{array}$$

where TP refers to true positives, FN refers to false negatives, TN refers to true negatives, and FP refers to false positives. Here, since we are looking for compounds that are not transported by BCRP (BCRP nonsubstrates) and we want to discard BCRP substrates, the previous expressions may be re-written as follows:

(3) $$\begin{array}{c} \text{Se}=\frac{\text{True}\;\text{nonsubstrates}}{\text{True}\;\text{nonsubstrates}+\text{False}\;\text{substrates}},\\ \text{Sp}=\frac{\text{True}\;\text{substrates}}{\text{True}\;\text{substrates}+\text{False}\;\text{nonsubstrates}}. \end{array}$$

By modifying the selection threshold from the lowest to the highest score provided by the individual models or the model ensemble, Se and Sp will evolve in opposite ways. Consequently, it is not possible to optimize both parameters simultaneously, and a tradeoff has to be found. ROC curves are a wide-used tool to assess and compare the performance of different models [46]. They are graphical plots of the sensitivity (true positives rate) versus 1 minus specificity (i.e., 1 less the false positives rate), for a binary classifier system, as its discrimination cutoff value changes. ROC curves provide a rational and user-friendly basis to balance type I and type II errors, selecting optimal models and optimal cutoff values. The AUC can be used for general comparison purposes of different models or methodologies. An ideal model will present an area under the ROC curve of 1 (equivalent to perfect classification, i.e., a sensitivity of 1 and a specificity of 1 for a given cutoff value), while random classification is represented by a line of slope 1 and corresponds to an area under the ROC curve of 0.5. Here, we have built ROC curves to compare the performance of the individual models developed and the performance of 2-model ensembles obtained through simple data fusion schemes. 

It has been pointed out that there is no general rule for balancing errors [46]. Balancing FP and FN depends on pragmatic considerations that are to be judged by the researcher [47]. We are interested in adopting a conservative attitude and developing highly specific models, that is, models capable of discarding practically all BCRP substrates. This is strongly related to our background: a small academic research group from a developing country with limited resources to invest in drugs acquisition and pharmacologic testing. Therefore, we will privilege Sp over Se. At the risk of losing some valuable scaffolds when applying our models in virtual screening campaigns, we will choose to avoid acquiring or synthesizing a drug candidate that, once send to pharmacological testing, will prove to be a FP (a drug that was predicted as a BCRP nonsubstrate but which is actually transported by BCRP). Taking into account that BCRP is characterized by broad substrate specificity (probably because, in part, of the existence of multiple binding sites in the protein), we have chosen to look for combinations of dfs that provide the lowest rate of FP in the external validation. Substrate polyspecificity indicates that it might be difficult to obtain a single model that is capable of identifying the entire set of BCRP substrates. We have combined the models by the very simple strategy of exhaustively looking for all the possible 2-model combinations of the models built from each of the pools of descriptors. The maximum (MAX operator) values among the values provided for each compound by the independent classifiers that compose the ensemble and the average (AVE) of the two values provided for each compound by the two independent models that compose the ensemble were used as data fusion schemes. 

### 2.4. Statistical Analysis of ROC Curves

It has been suggested that AUC of the ROC curves may not be the best parameter to compare different models, especially if the modeler is interested in comparing a particular region of the ROC curve instead of the entire curve (in our case, e.g., we are interested in the early zones of the curves corresponding to high Sp). A key requirement for the success of virtual screening is the ability of the combination of a scoring function to rank actives early in a large set of compounds; this has been referred as the early recognition problem [48]. Therefore, to compare the performance of the individual models and the model ensembles, we have applied, along with the total AUC comparison, partial AUC (pAUC) statistical analysis [49] and the calculation of other metrics (enrichment descriptors) that have been proposed to address the early recognition problem in virtual screening [48, 50, 51]. 

To fit ROC curves to the three sets of data, a nonparametric approach [52, 53] was used. The analysis was performed using *roc.jar *[54] and *pROC* packages for R [55].

The empirical Se and Sp were derived by dichotomizing the observed (empirical) values into positive or negative test-results for each observed cut point of the variable (*y*). As *y* varies over the observed values of the variables, the empirical ROC curve is defined as the discrete set of Se(*y*) and [1 − Sp(*y*)] values joined by straight lines [56]. The curve passes through point (0, 0) when *y* is larger than the maximum value observed, and it monotonically increases to the point (1, 1), as *y* decreases to the smallest observed value. To be informative, the curve should be above the 45° line at least for some of the values, where Se(*y*) is equal to 1 − Sp(*y*) [57]. 

In the nonparametric approach, the AUC is estimated by the trapezoid defined by the empirical set of Se(*y*) and [1 − Sp(*y*)] values, and its value is related to the *U* statistic for the two-sample Mann-Whitney/Wilcoxon rank-sum test [52, 53] and can be estimated accordingly, as well as the confidence intervals (CI) and the variance-covariance matrix [58]. Since the three ROC curves were built on variables observed on the same sample, they were paired, and therefore the null hypotheses of equal AUC's between two of them (*A* and *B* in the formula) were tested with a two-sample *z*-test:

(4) $$\begin{array}{c} z=\frac{\left({\text{AUC}}_{A}-{\text{AUC}}_{B}\right)}{\sqrt{{\mathrm{Var}}_{A}+{\mathrm{Var}}_{B}-2\cdot {\text{Covar}}_{AB}}}. \end{array}$$

For a pAUC estimation between two given Sp values, pROC package [56] was used.The numerical value of pAUC is estimated by the trapezoidal rule, whereas significance testing and confidence interval computation is performed by bootstrap with nonparametric stratified resampling (*n* = 2000). In stratified bootstrap, each replicate contains the same number of cases and controls than the original sample. Stratification is especially useful if one group has only little observations, or if groups are not balanced [59].

For the evaluation of the model performance, several enrichment descriptors were calculated for each model: area under the accumulation curve (AUCc) [60], enrichment factor (EF) [61], robust initial enhancement (RIE) [62] and Boltzmann-enhanced discrimination of ROC (BEDROC) [48]. All of these metrics were calculated with the *enrichvs *package for R [63].

### 2.5. Simulated Virtual Screening Campaign

An issue that emerges from using a reduced dataset (such as our 98-compound test set) is that the enrichment metrics derived exhibit a higher variance compared to significantly large datasets. Experiments conducted by Truchon and Bayly [48] show that the standard deviations associated with enrichment metrics such as ROC or AUCc are higher for small datasets and converge to a constant value when the size of the dataset increases.

The other problem is related to the high ratio of actives which mainly hinders the early recognition ability in what is known as the “saturation effect.” That is, for datasets with a high ratio of hits (in our case, BCRP nonsubstrates), once hit compounds saturate the early part of the ordered list, the enrichment metric cannot get any higher. To estimate in a more realistic way the utility of our model in a real virtual screening approach, we have dispersed our test set among 479 putative substrates acting as decoys. Such putative substrates are substrates of BCRP from other species rather than human or highly similar compounds to human BCRP substrates which have been retrieved from either ZINC database or Pubchem. This simulated database thus contains 27 known nonsubstrates among 550 known or putative substrates among 550 known or putative substrates; that is, the nonsubstrates ratio is less than 0.05, representing a more challenging set to assess the enrichment ability of our models. Note that some of these putative substrates (decoys) might actually be nonsubstrates; thus, the true performance of our models may be even higher than the one obtained through this simulated experiment. 

## 3. Results and Discussion

Based on the considerations exposed in the previous section, varying tolerance values between 0.1 and 0.5 and the maximum number of steps in the stepwise forward procedure between 10 and 15, we obtained 196-individual models from the 102-descriptor pools. Exhaustive 2-model combinations of these 196 models were performed by applying the MAX and AVE data fusion strategies.  Table 1 presents the statistics and validation outcome for those individual models that were later selected in the best 2-model ensembles. All the individual models that take part in the best 2-model combinations present an excellent cases per predictor ratio (from 13.7 to 32.8) indicating very low chance of overfitting. They present tolerances between 0.1 and 0.2, suggesting low pairwise correlation between the descriptors included in the models. As expected, the explanatory power of the randomized models is significantly below that of the actual (nonrandomized) model, since the correlation between the molecular structure and the modeled property is abolished when the dependent variable (in our case, the class label) is scrambled among the training set compounds. All models present conformation independent descriptors; thus, they may be applied to assist virtual screening campaigns, detecting (and discarding) potential BCRP substrates at the early stages of drug development projects, so that the retained candidates do not present low bioavailability or drug interactions issues due to their efflux transport by BCRP. The results of both the internal cross-validation and the external validation show adequate predictive power, especially considering the broad substrate specificity of BCRP. Nevertheless, these results have been remarkably improved when combining the individual models into 2-model ensembles, as shown in Table 2. 

The AVE data fusion scheme outperformed the use of the MAX operator. This is in line with previous reports empirically showing that consensus prediction by simple averaging of outputs of individual models is an efficient way to enhance predictive performances [64–69].

**Figure 2:**
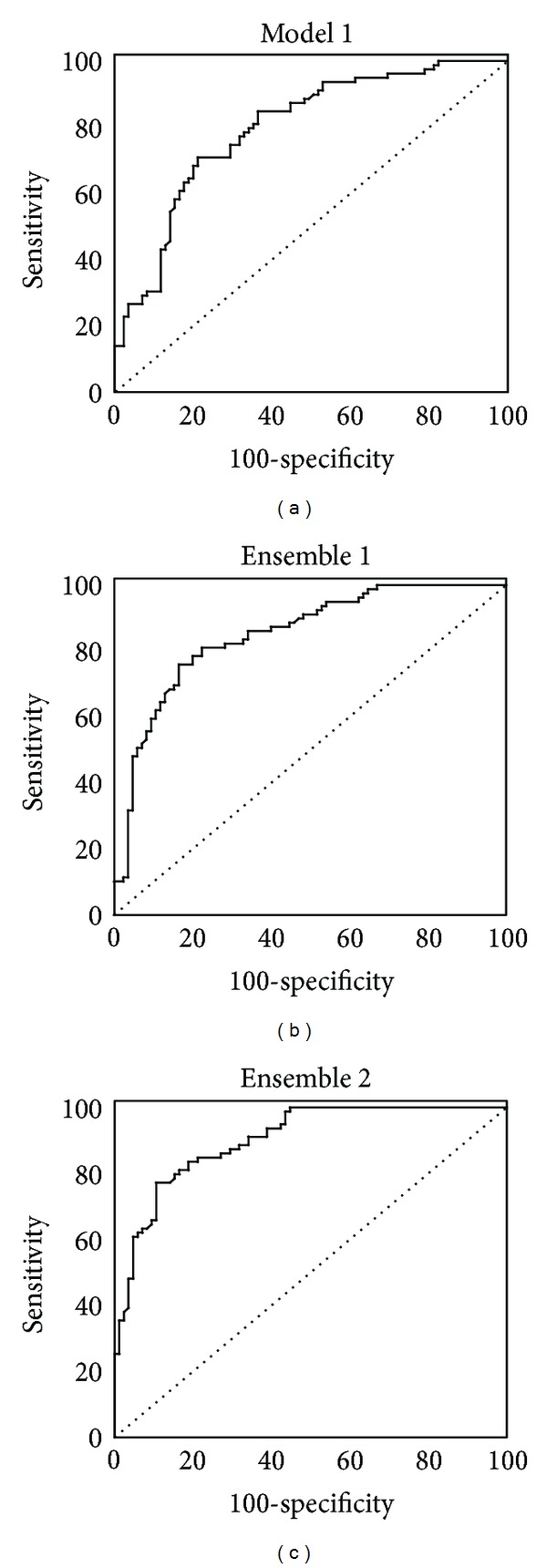
ROC curves of the training set for the best individual model plus the two best model ensembles.

The statistical comparison of the ROC curves (Figure 2) proved that ensemble 2 outperforms the best individual model (model 1) in the high Sp region in both the training set and the simulated 577-compound database. The results are summarized in Tables 3 and 4. We are particularly interested in the high Sp regions in order to assure discarding BCRP substrates in the early stages of the drug discovery process. The results confirm the utility of ensemble learning to identify ABC transporters substrates and nonsubstrates. ROC curves may also be applied to optimize the score threshold of the model or model ensemble, in order to select the best possible balance between Sp and Se, taking into account the active yield and other background-dependent considerations (e.g., budget, need to identify novel scaffolds).

## 4. Conclusion

We have developed a 2-model ensemble based on conformation independent Dragon descriptors for the identification of BCRP substrates and nonsubstrates. Since the descriptors incorporated into the models do not require preprocessing of the predicted chemical structure, the ensemble is particularly suitable to be applied in virtual screening campaigns of large chemical libraries in a highly efficient manner. Statistical comparison of ROC curves indicates that the best 2-ensemble model outperforms the best individual models generated. One should keep in mind that the broad substrate specificity of BCRP (and, in general, ABC transporters) makes it difficult to find a single linear relationship capable of accurately classifying substrates and nonsubstrates, a fact that justifies the application of more complex strategies such as ensemble learning or locally weighted regression methods. Unlike previously developed modeling efforts towards recognition of BCRP substrates and nonsubstrates, our models have been derived from a relatively large dataset which was split into representative training and test set through clustering analysis; the obtained training set presents a fair balance between the number of substrates and nonsubstrates. The studied ensemble is a potentially valuable tool to assist virtual screening and computer-aided drug design campaigns, as suggested by the outcome of the simulated virtual screening campaign. With the help of the constructed ROC curves, Sp and Se may be balanced to attend specific user requirements. 

## Supplementary Material

The structures of chemical compounds which compose the training and test sets are presented as Supplementary Material.

## Figures and Tables

**Table 1:** Features of the best individual model (Model 1) and the other individual models (models 2 to 4) that composed the two best 2-model ensembles.

Descriptors included	*F*	*P* value	Sp training set*	Se training set*	Overall accuracy training set*	Sp test set*	Se test set*	Overall accuracy test set*	Leave-group-out CV^1^	Randomization^2^
Model 1: *m*log⁡*P*2 (squared Moriguchi octanol-water partition coefficient), nCrR2 (no. of ring quaternary C(sp3)), JGI7 (mean topological charge index of order 7), nCONHR (no. of secondary amides (aliphatic)), nHAcc (no. of acceptor atoms for H-bonds (N O F)), and GGI8 (topological charge index of order 8).	8.04	<0.000000	79%	68%	74%	63%	74%	66%	70.4% (±11.9)	64.4% (±3.4)

Model 2: BEHm2 (highest eigenvalue no. 2 of Burden matrix/weighted by atomic masses), BELe2 (lowest eigenvalue no. 2 of burden matrix/weighted by atomic Sanderson electronegativities), Hy (hydrophilic factor), LAI (Lipinski alert index), LP1 (Lovasz-Pelikan index), BEHp1 (highest eigenvalue no. 1 of Burden matrix/weighted by atomic polarizabilities), SEigp (eigenvalue sum from polarizability weighted distance matrix), and VRA2 (average Randic-type eigenvector-based index from adjacency matrix).	7.52	<0.000000	75.3%	74.7%	75%	76%	66.7%	73.5%	67% (±15)	61.5% (±3.6)

Model 3: D/Dr11 (distance/detour ring index of order 11), nCONHR, nCO (no. of ketones (aliphatic)), X0Av (average valence connectivity index chi-0), nCaH (no. of unsubstituted aromatic C(sp2)), Xt (total structure connectivity index), PW4 (path/walk 4-Randic shape index), D/Dr12 (distance/detour ring index of order 12), T(O..O) (sum of topological distances between O..O), nNHRPh (no. of secondary amines (aromatic)), SPI (superpendentic index), and Rww (reciprocal hyperdetour index).	10.39	<0.000000	83.5%	83.5%	83.5%	73.2%	74%	73.5%	81.2% (±11.3)	62.4% (±5.1)

Model 4: *m*log⁡*P*2, JGI7, SRW10 (self-returning walk count of order 10), piPC02 (molecular multiple path count of order 02), and Hy.	6.56	<0.000014	63.3%	70.6%	67%	77.5%	70.4%	75.5%	64.8% (±13.6)	58.3% (±4.05)

*Considering zero as a cutoff value between substrates and non-substrates. This threshold may be later optimized through ROC curves analysis to provide a background-dependent optimal balance between Sp and Se.

^1^Results are presented as the average result for the folds ± the standard deviation.

^2^Results are presented as the average performance of the randomized models ± the standard deviation.

**Table 2:** Features of the best individual model (Model 1) and the best ensembles selected.

Model/ensemble	AUC ROC curve training set	AUC ROC curve test set	Sp training set*	Se training set*	Overall accuracy training set*	Sp test set*	Se test set*	Overall accuracy test set*
Model 1	0.796	0.748	78.8%	68.3%	74%	63.4%	74%	66%
Ensemble 1	0.850	0.785	83.5%	74.7%	79%	70.4%	74%	71.4%
Ensemble 2	0.902	0.804	84.7%	79.7%	82%	76%	70.4%	74.5%

*Considering zero as a cutoff value between substrates and non-substrates.

**Table 3:** Results of the calculation of the total and partial areas under ROC curve for the best individual model and the 2 best 2-model ensembles.

	Model 1	Ensemble 1	Ensemble 2
Training set			
Total ROC curve AUC (95% CI)	0.7964 (0.7284–0.8643)	0.8503 (0.7917–0.9089)	0.9022* (0.8578–0.9466)
Partial ROC curve AUC (±SD)			
From 1 to Sp = [1 to 0.70]	0.1612 (±0.0199)	0.1877 (±0.0199)	0.2218 (±0.0163)*
From 1 to Sp = [1 to 0.75]	0.1252 (±0.0171)	0.1459 (±0.0178)	0.1771 (±0.0145)*
From 1 to Sp = [1 to 0.80]	0.0917 (±0.0147)	0.1059 (±0.0149)	0.1337 (±0.0122)^†^

Simulated 577-compound database			
Total ROC curve AUC (95% CI)	0.7321 (0.6413–0.8229)	0.7357 (0.6418–0.8297)	0.7707 (0.6746–0.8669)
Partial ROC curve AUC (±SD)			
From 1 to Sp = [1 to 0.70]	0.1035 (±0.0213)	0.1127 (±0.0223)	0.1421 (±0.0223)
From 1 to Sp = [1 to 0.75]	0.0708 (±0.0183)	0.0794 (±0.0189)	0.1075 (±0.0208)^†^
From 1 to Sp = [1 to 0.80]	0.0458 (±0.0140)	0.0504 (±0.0148)	0.0765 (±0.0162)^†^

*The value is different from the best individual model (model 1) (*P* < 0.001).

^†^The value is different from the best individual model (model 1) (*P* < 0.01).

**Table 4:** Results of the enrichment parameters calculation for the best individual model and the best 2-model ensemble.

	Model 1	Ensemble 2
Training set		
Accumulation curve AUC (AUCc)^‡^	0.6458	0.6938
Enrichment factor (EF)	1.9294	1.9294
Robust initial enhancement (RIE)	1.8338	1.9261
Bedroc	0.9505	0.9983
Simulated 577-compound database		
Accumulation curve AUC (AUCc)^‡^	0.7212	0.7581
Enrichment factor (EF)	2.9630	5.9259
Robust initial enhancement (RIE)	2.9455	4.6663
Bedroc	0.2268	0.3593

^‡^It verifies that ROC AUC = AUCc/*R*_i_ − *R*_a_/(2∗*R*_i_), where *R*_i_ and *R*_a_ are the ratios of inactives and actives, respectively.
