# Supplementary figures and images for: Development of Conformation Independent Computational Models for the Early Recognition of Breast Cancer Resistance Protein Substrates
Source: Biomed Res Int. 2013 Aug 1;2013:863592. doi: 10.1155/2013/863592 (PMC3747366; doi:10.1155/2013/863592)

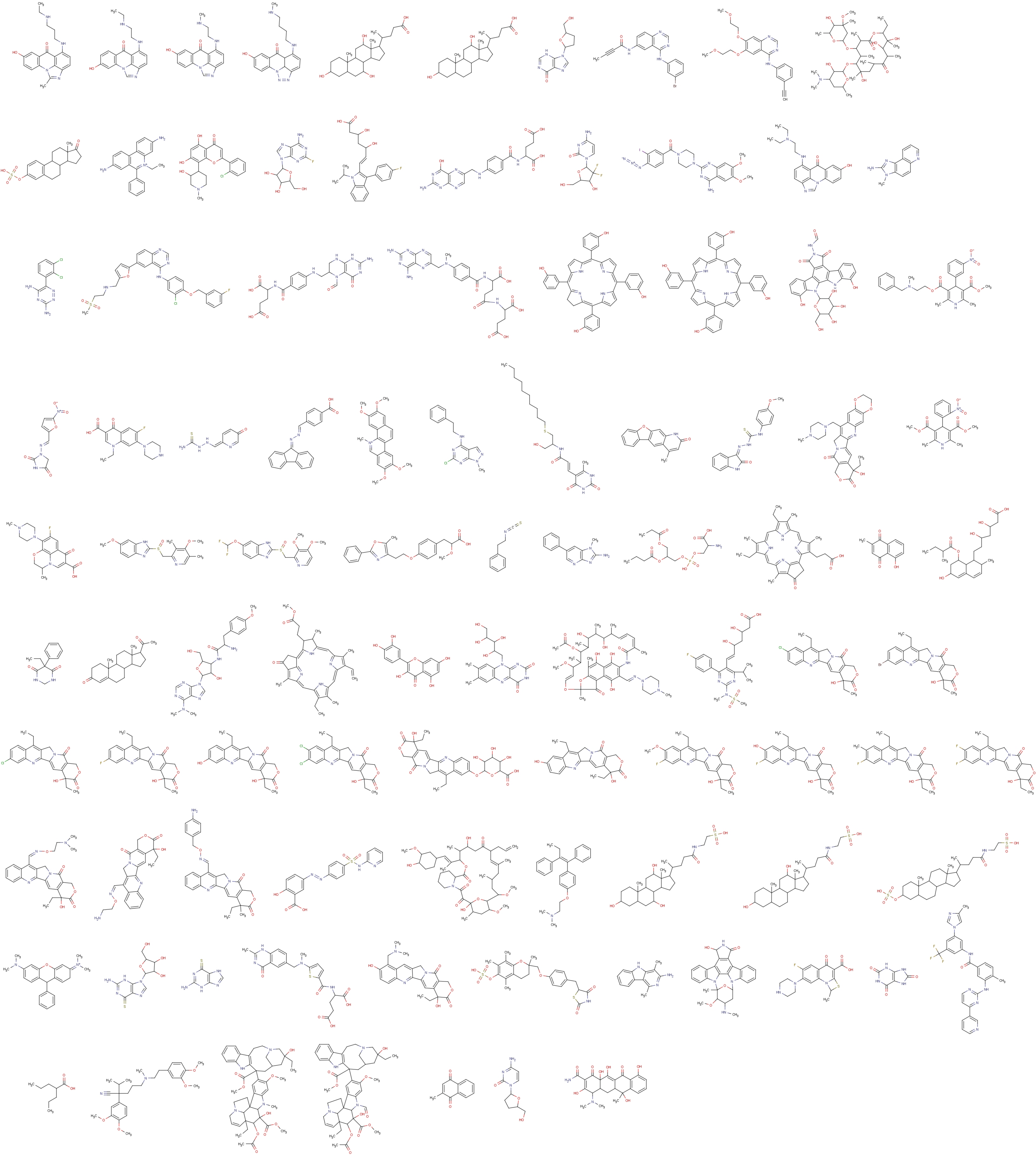

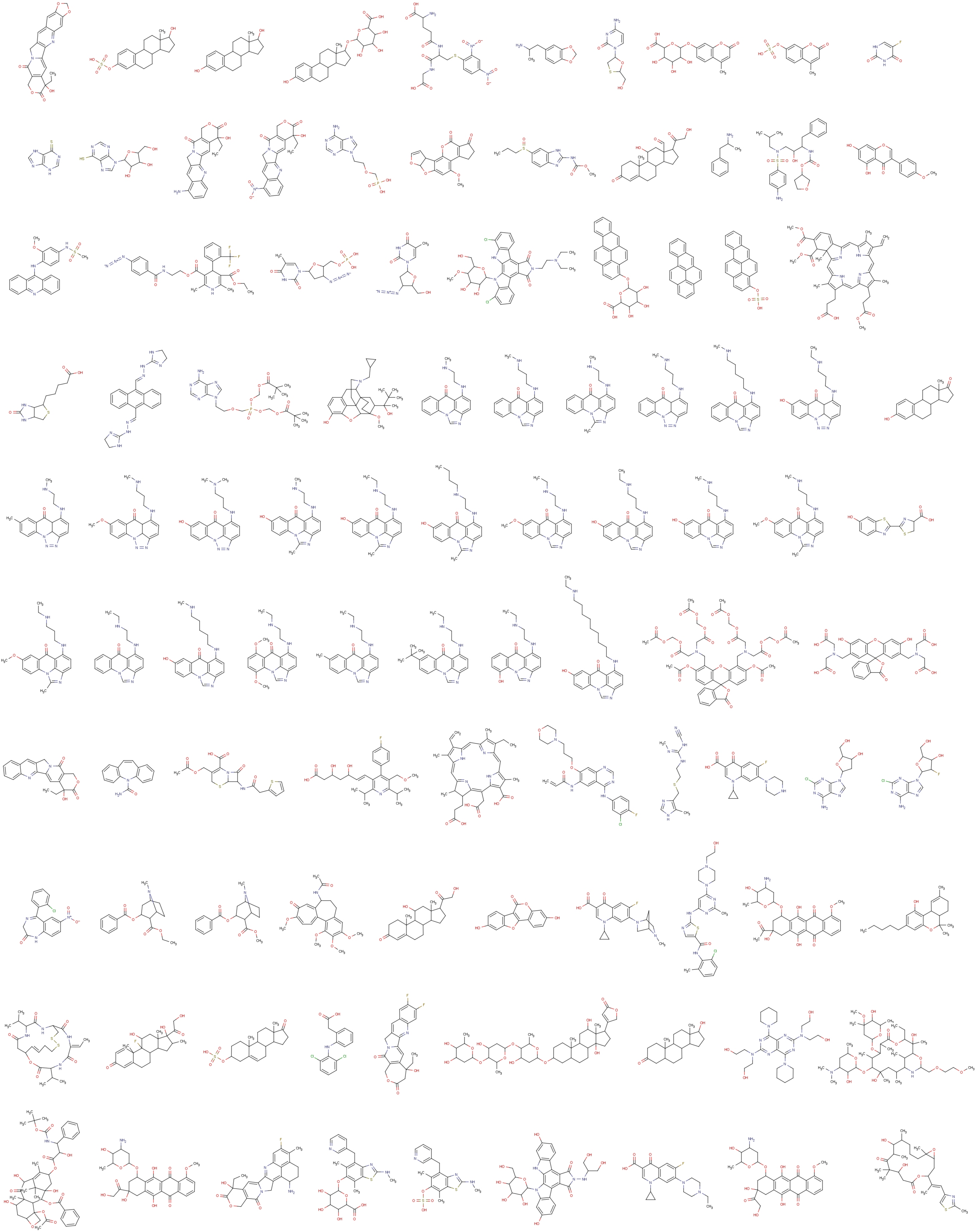

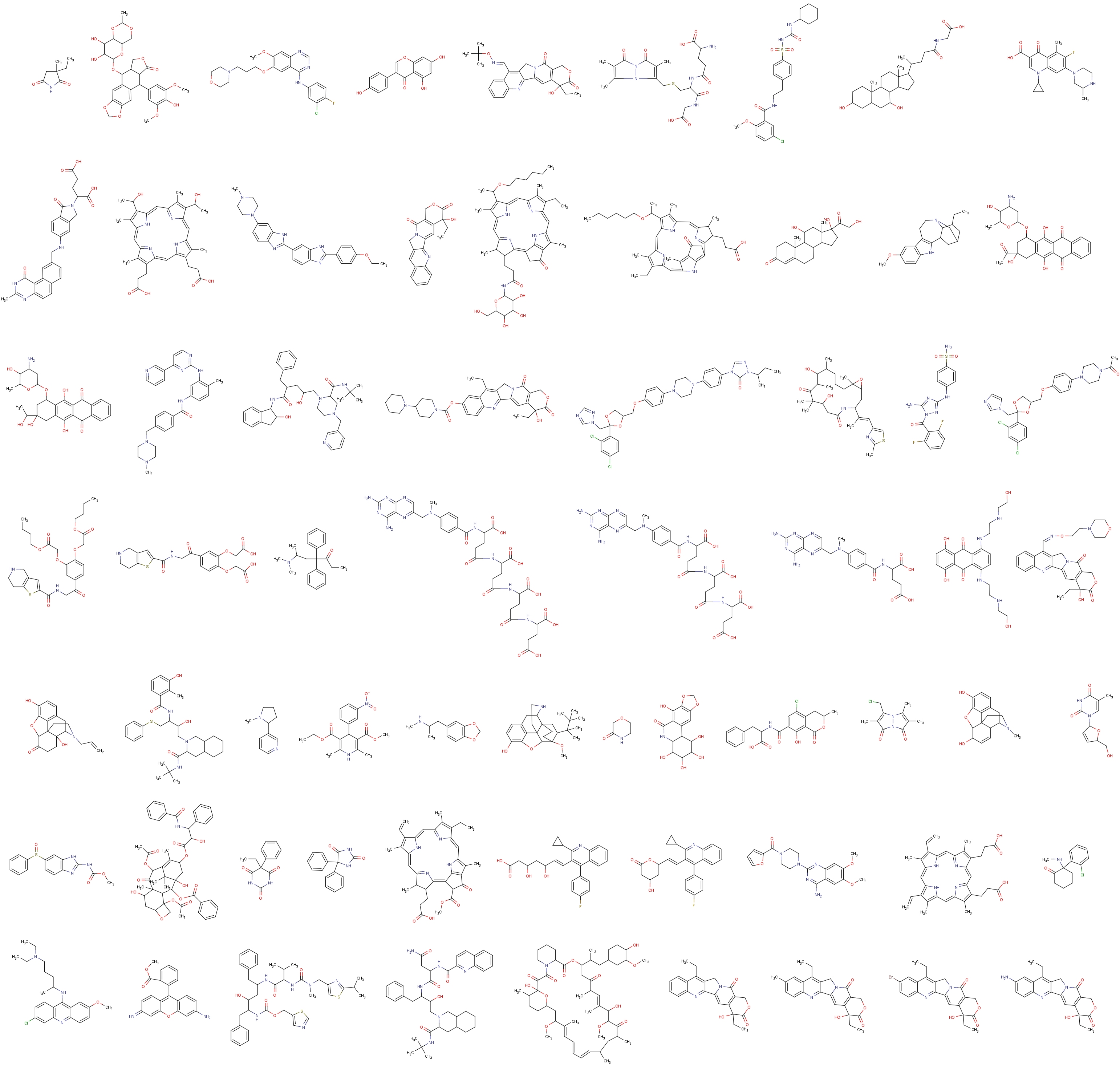

Supplement: Supplementary file 1 — The structures of chemical compounds which compose the training and test sets are presented as Supplementary Material. [file 863592.f1.pdf]
